# Telomerase reverse transcriptase and neurodegenerative diseases

**DOI:** 10.3389/fimmu.2023.1165632

**Published:** 2023-03-29

**Authors:** Xin Yu, Meng-Meng Liu, Cai-Yun Zheng, Yu-Tong Liu, Zhuo Wang, Zhan-You Wang

**Affiliations:** Key Laboratory of Major Chronic Diseases of Nervous System of Liaoning Province, Health Sciences Institute of China Medical University, Shenyang, China

**Keywords:** telomerase reverse transcriptase, aging, neurodegenerative diseases, T cell, nervous system

## Abstract

Neurodegenerative diseases (NDs) are chronic conditions that result in progressive damage to the nervous system, including Alzheimer’s disease (AD), Parkinson’s disease (PD), Huntington’s disease (HD), and Amyotrophic lateral sclerosis (ALS). Age is a major risk factor for NDs. Telomere shortening is a biological marker of cellular aging, and telomerase reverse transcriptase (TERT) has been shown to slow down this process by maintaining telomere length. The blood-brain barrier (BBB) makes the brain a unique immune organ, and while the number of T cells present in the central nervous system is limited, they play an important role in NDs. Research suggests that NDs can be influenced by modulating peripheral T cell immune responses, and that TERT may play a significant role in T cell senescence and NDs. This review focuses on the current state of research on TERT in NDs and explores the potential connections between TERT, T cells, and NDs. Further studies on aging and telomeres may provide valuable insights for developing therapeutic strategies for age-related diseases.

## 1 Introduction

Neurodegenerative diseases (NDs) are a group of neurological disorders characterized by the loss of neurons or myelin sheaths, leading to progressive motor and cognitive impairment. The most common forms of these diseases include Alzheimer’s Disease (AD), Parkinson’s Disease (PD), Huntington’s Disease (HD), and Amyotrophic Lateral Sclerosis (ALS). The development of NDs is linked to a range of factors, including aging, oxidative stress, inflammation, mitochondrial dysfunction, and protein aggregate accumulation (1–5). Aging is the most significant risk factor and is characterized by metabolic dysfunction, mitochondrial dysfunction, and telomere wear (6). Understanding and addressing the aging process has the potential to improve many age-related conditions, including neurodegenerative diseases. Immune senescence, marked by a decline in T cell immune function, is a common feature of aging. However, it is not very clear whether immune senescence is associated with NDs, and T-cell aging drives aging of other non-immune organs (7). Additionally, Natural Killer (NK) cells increase progressively in the brain with age. Studies have found that clearing immune cells in the aging brain promotes neuroblastoma cell survival and improve cognitive function (8). T-lymphocyte senescence is primarily caused by shortened telomere length or impaired telomerase function (9). Telomeres, widely recognized as hallmarks of aging, have also been implicated in the process of neurodegeneration in neurodegenerative diseases (10, 11).

Telomeres are repetitive DNA sequences (TTAGGG)n located at the ends of eukaryotic linear chromosomes. They protect cells during replication, prevent DNA damage, and regulate the cell division cycle through their telomeric DNA and telomere binding proteins (12, 13). As cells divide, telomeres gradually shorten, but when this reaches a critical point, telomerase can be activated to lengthen telomeres, allowing cells to survive a crisis state or replicate indefinitely (14, 15). Telomerase activity, which is essential for maintaining telomere length, is primarily composed of the Telomerase reverse transcriptase (TERT) protein, telomerase RNA component (TERC), and telomerase-related catalytic proteins (16, 17). TERT is the primary determinant of telomerase activity, and it is normally found in germ cells, neurons, and stem cells, but is inactive in somatic cells (18–22). Exploring telomere biology offers the potential to search for effective strategies for treating neurodegenerative diseases. The expression of TERT can be influenced by various factors, such as cycloastragenol (CAG), a telomerase activator, which has been shown to promote TERT expression in rat neurons (21–23). GRN510, a novel small molecule compound, is another example of a telomerase activator that has protective effects against neurological diseases by upregulating TERT expression (24). TERT levels remain constant during embryonic development, despite decreasing telomerase activity (25). TERT has been found to play multiple roles, including neuronal protection, anti-inflammation, antioxidant, tumor suppression, immunomodulation, and reduction of toxic proteins (26). TERT can also reduce oxidative stress by transporting from the nucleus into mitochondria and reducing the production of reactive oxygen species (ROS) (27, 28) Upregulated TERT expression in microglia has also been shown to inhibit the production of inflammatory factors and have anti-inflammatory effects (29).

Oxidative stress is a significant contributor to the development of neurodegenerative diseases and TERT has been found to play a role in addressing these conditions. As such, TERT has emerged as a promising strategy for the treatment of age-related NDs (30, 31) Research has shown that telomerase activation promotes lymphocyte proliferation and slows down cellular senescence, while TERT upregulation prevents or reverses tissue and organ degeneration and premature aging in mice (11). Neurodegenerative diseases are characterized by an altered balance of oxidation-reduction, leading to disruptions in cellular signaling pathways and the regulation of the immune response and inflammatory processes through ROS (32). In NDs, the intracellular accumulation of amyloid β protein and neuronal death is accompanied by increased Th1-Th17 responses and decreased Th2 responses (33). In the central nervous system, pro-inflammatory factor-mediated neuroinflammation in glial cells can be regulated by peripheral T cells, while CD8+ T cells and CD4+ T cells are capable of inducing significant neuronal death (34, 35). IL-4-deficient mice with inflammatory myeloid cells exhibit cognitive deficits, but wild-type T cells can reverse this effect (36).

In conclusion, TERT has the potential to play a role in NDs by regulating oxidative stress and inflammatory responses, which can impact Treg cells. The progress of research on the function of TERT in NDs is discussed in this paper.

## 2 TERT and T cells

The role of T lymphocytes in the immune system and the relationship between TERT and T cells have been widely studied. T lymphocytes are a type of immune cell that mature in the thymus and are the main component of lymphocytes. T lymphocytes are the most numerous and complex in terms of function, and aging can decrease and abnormalize the body’s immune function by affecting T lymphocytes (37). Regulatory T cells (Treg cells) are a subset of T cells that play an important role in maintaining self-tolerance, suppressing autoimmunity, and regulating inflammation in neurodegeneration. It has been shown that Treg cells are involved in an adaptive response when the cellular environment is altered, in order to maintain the tissue internal environment’s homeostasis (38).

TERT is an antigen that is closely related to the development of immunotherapies, and T cells are at the center of the “immune surveillance” process (39). The expression of TERT has been found to be closely associated with immunosuppressive features in the form of Th2 cells, Treg cells, CD56dim natural killer cells, and myeloid-derived suppressor cells (40). Additionally, overexpression of the TERT gene has been shown to enhance the proliferative capacity of T cells, which provides a potential therapeutic strategy for diseases such as tumors (41). TERT is expressed in all stages of tumor differentiation and is a crucial target for immunotherapy, with CD4 T cells playing a role in combating TERT in cancer (39). Inflammation affects the body’s immune response, and numerous studies have confirmed that several inflammatory factors, including IL-2 and IL-6, can promote the expression of telomerase in T cells and keep it at high levels (42). The NF-κB signaling pathway is an important pathway that regulates the inflammatory response, and its downstream target genes include inflammatory factors such as IL-6, IL-8, and TNF-α, the overexpression of which leads to immune dysfunction. TERT has a forward feedback regulation with the NF-κB signaling pathway, allowing it to regulate the inflammatory immune response of cells (43). The transcriptional regulation of NF-κB by TERT has been shown to be responsible for the expression of genes in metabolic syndrome and the regulation of immune cell function (44, 45). Additionally, TERT gene-transduced T cells have an enhanced ability to resist oxidative stress (46), and there is evidence that T cells overexpressing TERT have a stronger rate of proliferation and are not malignant (47). Importantly, siRNA mediated TERT knockdown increased cellular ROS levels, while TERT overexpression inhibited endogenous ROS production (48). *In vitro* experiments showed that reducing TERT activity promoted oxidative stress, leading to cell death (26, 49). With aging, telomere length shortening occurs in various organs, causing more cells to enter the senescent phase and lose their proliferative capacity (50).

The role of T cells in the brain is increasingly being recognized and as the relationship between aging and neurodegenerative diseases becomes more evident, there is growing interest in studying T cells for potential therapeutic insights into these aging-related conditions. Although there is limited research on TERT and T cells, the potential for further exploration is significant.

A recent study found that T cells play an important role in the brain and considering the relationship between aging and neurodegenerative diseases (51), it is not difficult to imagine that a deeper study of T cells may provide important therapeutic clues for aging-related diseases, including neurodegenerative diseases. Despite the relatively few studies related to TERT and T cells, there is great value for exploration.

## 3 Role of TERT in neurodegenerative diseases

As previously mentioned, the aging process is closely tied to the development of neurodegenerative diseases. This is often characterized by oxidative stress, neuroinflammation, and an altered immune response. Research has shown that prolonged exposure to pro-oxidants leads to the production of reactive oxygen species (ROS) which, in turn, causes cellular damage through inflammation and mitochondrial dysfunction. In short, there is an interaction between oxidative stress and inflammatory responses, and the inflammatory factors produced by the body can potentially cause an immune response that can affect the progression of NDs. This results in increased expression of IL-17 and Syntaxin5 in the hippocampus of the brain, leading to cognitive impairment (32). Given the anti-aging and antioxidant effects of TERT, it is essential to further examine the relationship between TERT and various neurodegenerative diseases.

### 3.1 TERT and AD

Alzheimer’s disease (AD) is a common neurodegenerative disorder that increases in incidence and prevalence with age (52–55). It is predominantly found in individuals over 65 years old (56). The underlying pathological features of AD are the presence of large numbers of senile plaques (SPs) formed by beta-amyloid peptide (Aβ) deposits outside the neuros (57) in the brain, as well as neurofibrillary tangles (NFTs) formed by tau-hyperphosphorylation (58, 59), oxidative stress (60), and alterations in cholinergic signaling (61). Studies have demonstrated that oxidative stress plays a critical role in the development and progression of AD, promoting Aβ deposition, Tau protein hyperphosphorylation, metal ion imbalances, synaptic and neuronal loss, and mitochondrial dysfunction (62, 63). Thus, targeting oxidative stress holds great potential as a therapeutic strategy for AD.

Studies have shown that patients with Alzheimer’s disease (AD) have elevated levels of CD4+/CD8+ T cells in peripheral blood, which is related to the permeability of the blood-brain barrier, which increases as the disease progresses, promoting the infiltration of T cells into the brain tissue (64–66). There are conflicting results regarding the role of T cells in AD, with some studies suggesting a neuroprotective role for Treg cells and an improvement in cognitive function with higher levels of Treg cells and IL-35 (67). On the other hand, elevated levels of TEMRA CD8 T cells, which are cytotoxic and secrete inflammatory molecules, have been found to impair cognitive performance in AD patients (68, 69). Furthermore, peripheral T cell activation and the subsequent release of pro-inflammatory factors such as IL-6, tumor necrosis factor α (TNFα), and IL-1, have also been linked to AD pathology (70). While multiple studies have suggested a role for T cells in AD development, their specific mechanisms of action and impact on the disease remain unclear.

The length of telomeres, which serve as a marker of cellular aging, are critical to normal brain function (71, 72). Given this importance, the potential benefits of TERT in preventing and treating Alzheimer’s disease (AD) have garnered significant attention. AD is predominantly seen in the elderly population, but studies have shown that TERT levels in the brains of AD patients remain unchanged with disease progression (73). TERT has been found to have multiple biological roles in adult brain neurons, including the accumulation in the mitochondria of AD patients’ brains and the prevention of neuronal damage from pathological proteins (74). Additionally, TERT has been shown to alleviate memory impairment in AD (75). AGS treatment was found to temporarily increase TERT gene expression in hippocampal primary cell cultures, either with or without Aβ, and ultimately protect neurons from Aβ-induced neuronal degeneration by increasing expression of neurotrophic factors, neuronal plasticity genes, and activating the Wnt/beta-catenin pathway (76). The telomerase protein TERT confers neuronal resistance to pathological tau by reducing the production of oxidative species and improving mitochondrial function rather than altering tau protein (77). Furthermore, neurons treated with TERT inhibitors were found to be more susceptible to Aβ damage, while TERT was able to protect neurons from Aβ-induced apoptosis in an experimental model of AD (78, 79).In conclusion, TERT may be a promising target for AD treatment and provide a new direction for research. However, its specific mechanisms of action require further investigation.

PD is a neurodegenerative disease that is second only to AD in prevalence worldwide (80) The aging population is leading to a growing number of PD patients (81). PD primarily affects the elderly, causing progressively worsening and irreversible symptoms, which can be divided into motor and non-motor symptoms (82). Pathologically, PD is characterized by the loss of neurons in the substantia nigra, the presence of eosinophilic protein deposits in the cytoplasm, and a reduction in dopaminergic neurons in the striatum of patients (83, 84). Lipid peroxidation, protein carbonylation, and 8-hydroxyguanosine have been found in the dopamine-producing neurons of PD patients (85). Neuroinflammation is also considered to play a crucial role in the development of PD. In PD model mice, the protective effect of the substantia nigra and striatum is mainly due to activated Treg cells (86). Pro-inflammatory cytokines such as tumor necrosis factor (TNF-α), interferon (IFN)-γ, interleukin (IL)-1β and IL-6 have been found to be elevated in the nigrostriatal region of PD patients (87). In the peripheral blood of PD patients, the levels of naive T cells and anti-inflammatory regulatory T cells are reduced, and the ratio of IFN-γ to IL-4-producing T cells is increased, creating a pro-inflammatory environment (88). α-Syn-reactive T cells have been found in PD patients, and they are most abundant in those with motor PD (89). Innate and adaptive immune changes occur in both the brain and periphery in PD, including adaptive T-lymphocyte responses (90). Additionally, a reduction in replicative senescence of CD8+ T cells in the early stages of PD has been shown to promote PD progression (91).

There is evidence that telomerase activators can have neuroprotective effects by increasing TERT expression in the brains of PD model mice and enhancing their locomotor abilities (92). Studies have shown that after CAG treatment in PD mice, there was a significant reduction of alpha-synuclein in the hippocampus and cortex, and a significant improvement in the mice’s motor function, suggesting that TERT can alleviate the symptoms of PD by inhibiting the aggregation of alpha-synuclein (74). It has been shown that the presence of estrogen receptor (ER) control elements in the promoter sequence of TERT elements, the integrity of ER is required for the regulation of hTERT, and the expression and distribution of ER are simultaneously regulated by the ratio of androgen to estrogen in the internal environment (93). TERT has a known association with estrogen, and the results of the rotating bar test after CAG treatment in PD model mice showed that motor function was significantly improved in females compared to males, which may be due to the protective effect of TERT (94). After 1‐Methyl‐4‐phenyl‐1,2,3,6‐tetrahydropyridine (MPTP) intraperitoneal injection in wild mice and TERT knockout mice, it was found that TERT-deficient mice displayed more severe symptoms, demonstrating that TERT-deficient mice are more susceptible to the neurotoxic effects of MPTP (95). Additionally, knockdown of TERT in the hippocampal dentate gyrus impaired the formation of spatial memory in mice (96). These findings suggest that the pathogenesis of PD could be improved by targeting TERT.

### 3.3 TERT and HD

Huntington’s disease (HD) is a debilitating neurodegenerative disorder that is characterized by the progressive decline of cognitive and motor functions, psychiatric disorders, and metabolic abnormalities (97). It typically presents in individuals between the ages of 30 and 40 years, although 5% of patients may present before the age of 21 years (97, 98). The disease is caused by the repetitive amplification of the first exon of the HTT gene, which encodes the Huntington protein. Research has shown that leukocyte telomere length is shorter in HD patients compared to healthy individuals, suggesting that telomere shortening may play a role in the pathogenesis of HD (99). The peripheral immune dysfunction in Huntington’s disease appears to be primarily mediated by the innate immune system, rather than the adaptive immune system (100). Additionally, therapies that decrease T cell-driven inflammation have been shown to delay or prevent the onset of HD (101). The results of relevant investigations have also suggested a potential association between telomere shortening and HD, which may be related to oxidative stress (102). As TERT is known to be a key component in maintaining telomere length and has antioxidant effects, it may be a potential target for alleviating HD and warrant further study.

### 3.4 TERT and ALS

Amyotrophic Lateral Sclerosis (ALS) is a type of motor neuron disease that affects mostly sporadic patients, with around 10% of cases being hereditary (103). The disease typically begins to develop between the ages of 40 and 60, with a high incidence rate (104). ALS is a rapid and fatal neurodegenerative disease characterized by the death and dysfunction of motor neurons in the patient’s brainstem, cerebral cortex, and spinal cord (105). Although the disease is influenced by multiple factors, it is currently incurable. Some studies have shown that immune system-related genes can play a role in ALS pathogenesis, including Treg cells that have a protective effect. For example, Foxp3, TGF-β, IL-4, and Gata3 mRNA levels were found to be reduced in patients with rapidly progressing ALS (106). In addition, research in mice expressing SOD1 suggests that deleting the gene in astrocytes or bone marrow compartments slows ALS progression (107). Moreover, peripheral immune cells have been shown to contribute to neurodegeneration (108). The study also found that the presence of clonally expanded TEMRA CD8 T cells in the peripheral blood of ALS4 patients may serve as a potential biomarker for exploring the disease’s pathogenesis (109). Furthermore, the levels of CD4+ T lymphocytes in the blood of ALS patients have been linked to evaluating cognitive impairment and age (110). TERT expression was found to be reduced in ALS patients (111) and a study showed that the AGS-499 compound improved motor neuron survival and delayed ALS progression in mice by promoting TERT expression in the brain in a dose-dependent manner (112). This suggests that TERT could be a promising target for the development of ALS therapies.

In conclusion, neurodegenerative diseases such as AD, PD, HD, and ALS can impact individuals at different life stages and pose a significant burden on affected individuals and society. Despite extensive research, current treatments for these diseases are limited and ineffective, making it crucial to better understand the underlying pathological mechanisms and causes of neurodegenerative diseases. Upon reviewing the literature, it is evident that there is a scarcity of studies that focus on the role of TERT in neurodegenerative diseases, especially in the context of immune aging. Thus, exploring the potential connection between TERT’s antioxidant effects and T cell senescence could be a promising avenue for future research and could offer new insights into the treatment of neurodegenerative diseases.

## 4 Conclusion and future prospects

**Figure 1:**
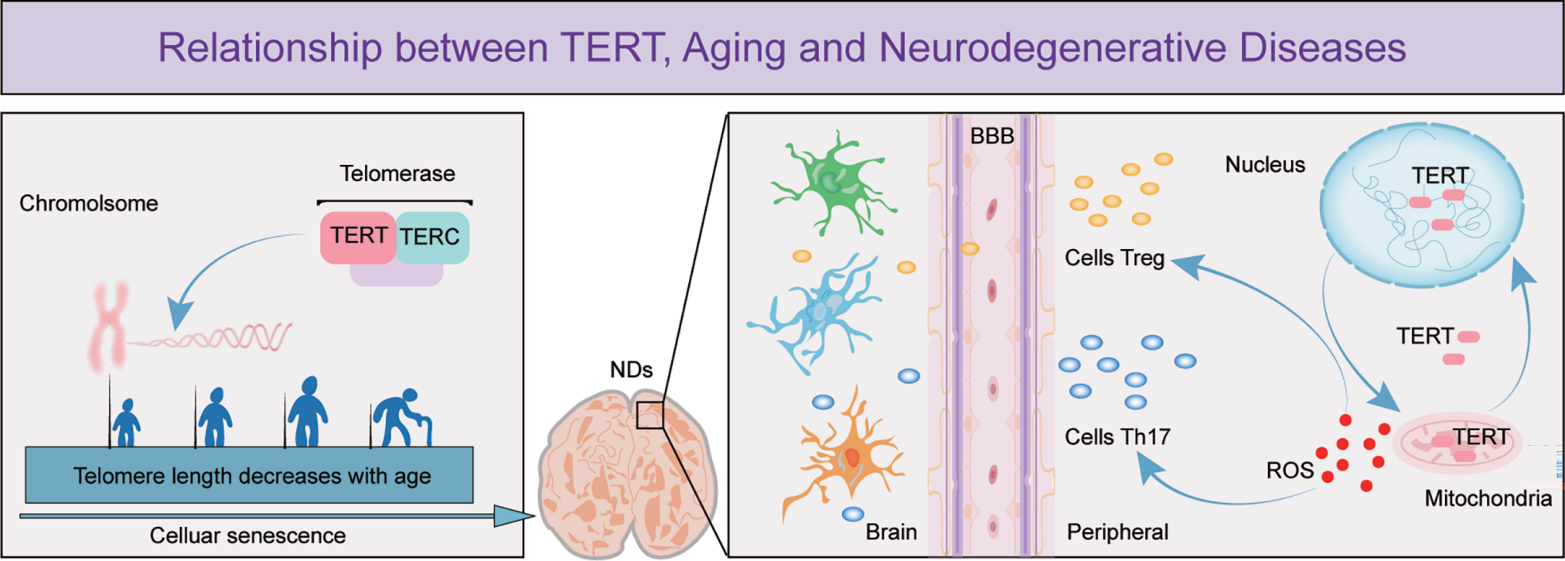
The figure depicts the important role of TERT in telomere maintenance and regulation of T cell effects in neurodegenerative diseases. The figure shows that as cells age, telomeres shorten in length and TERT, an essential component of telomerase, has a crucial function in preserving telomere length. Cellular senescence is a leading cause of neurodegenerative diseases and research has demonstrated that as these diseases progress, the permeability of the blood-brain barrier increases, enabling more peripheral T cells to enter the brain. TERT helps to regulate T cell expression by maintaining the optimal balance of intracellular oxidative stress, thereby exerting a neuroprotective effect.

The search for effective solutions to neurodegenerative diseases has become increasingly urgent due to the slow progress in research on these diseases. Aging is the main risk factor and the pathological changes it causes are irreversible. T cell senescence has emerged as an important area of research due to its role in aging and the recent discovery of its role in neurodegenerative diseases. TERT, which maintains telomere length and delays cellular senescence, has been shown to have neuroprotective effects (**Figure 1**). However, there is limited research on the relationship between T cell senescence and neurodegenerative diseases, which has led to a growing interest in exploring the potential link between TERT and T cells in this context. This review summarizes the relevant roles of TERT and the potential relationship between T cell senescence and neurodegenerative diseases, in order to provide insights into the pathogenesis of these diseases and pave the way for the development of new therapeutic agents.

## Author contributions

XY and M-ML wrote the manuscript. T-YL and ZW contribution to the figure. Y-CZ reviewed the manuscript. XY and Z-YW edited and finalized the manuscript. All authors contributed to the article and approved the submitted version. 
